# Development and validation of a theoretical test for ultrasound-guided biopsy competence in the head and neck

**DOI:** 10.1007/s00405-026-10502-7

**Published:** 2026-08-03

**Authors:** Kasper Daugaard Larsen, Lars Konge, Jacob Melchiors, Lisa A. Orloff, Merry E. Sebelik, Andrew McQueen, Gitta Madani, Jacob Pohl Stangerup Nielsen, Tsung-Lin Yang, Julian Kuenzel, Leila J. Mady, Tina Klitmøller Agander, Tobias Todsen

**Affiliations:** 1 Department of Otorhinolaryngology, Head and Neck Surgery and Audiology, Rigshospitalet, Copenhagen, Denmark; 2 Copenhagen Academy for Medical Education and Simulation, University of Copenhagen and The Capital Region of Denmark, Copenhagen, Denmark; 3 Department of Otolaryngology-Head & Neck Surgery, Stanford University School of Medicine, Stanford, CA USA; 4 Department of Otolaryngology-Head and Neck Surgery, Emory University School of Medicine, The Winship Cancer Institute at Emory University, Georgia, USA; 5 Department of Radiology, Freeman Hospital Newcastle upon Tyne Hospitals NHS Foundation Trust, Newcastle upon Tyne, UK; 6 Department of Radiology, Imperial College Healthcare NHS Trust, London, UK; 7 Department of Otolaryngology, National Taiwan University Hospital, National Taiwan University College of Medicine, Taipei, Taiwan; 8 Department of Otorhinolaryngology, Head and Neck Surgery, University Hospital Regensburg, Regensburg, Germany; 9 Department of Otolaryngology – Head and Neck Surgery, The Johns Hopkins School of Medicine, Baltimore, USA; 10 Department of Pathology, Rigshospitalet, Copenhagen, Denmark; 11 Department of Applied Mathematics and Computer Science, Technical University of Denmark, Lyngby, Denmark

**Keywords:** Ultrasound-guided biopsy, Competency assessment, Delphi process, Medical education, Head and neck, Validity evidence

## Abstract

**Purpose:**

This prospective study is designed to test the validity of a multiple-choice question (MCQ) test in assessing theoretical knowledge of ultrasound-guided fine-needle aspiration biosy and core-needle biopsy of the head and neck.

**Methods:**

Seven international experts reached consensus on the MCQ test using a modified Delphi process. After consensus, the MCQ was tested on novices (*n*=59) and experienced specialists (*n*=17). Item analysis was performed to evaluate difficulty and discriminatory ability, Cronbach’s α was calculated to assess internal consistency reliability, and group differences were compared using ANOVA and t-tests. The contrasting groups’ standard setting method was applied to set a pass/fail standard and thereafter, participants in a head and neck ultrasound course (*n*=27) took the test to assess the consequences.

**Results:**

Two Delphi rounds yielded a 23-item MCQ test that reflected essential knowledge for ultrasound-guided biopsies. Two poorly performing items were removed following item analysis, resulting in a 21-item test with good reliability, Cronbach’s α=0.82. Experienced clinicians scored significantly higher (mean 20.0±0.9) than both course participants (17.3±2.8) and novices (13.5±3.6, *p*<0.001). A pass/fail score of 18/21 (≈86%) was established as the cut-off. At this standard, 100% of experts passed the test while only 13.6% of novices and 63% of course participants achieved a passing score.

**Conclusion:**

An MCQ test of theoretical knowledge for ultrasound-guided head and neck biopsies was developed with evidence of validity across all five sources in Messick’s framework. The test demonstrated strong content validity through expert consensus, good internal structure, significant discrimination between experience levels, and a defensible pass/fail standard. At present, the instrument should be interpreted as a theoretical knowledge assessment that may complement, but not replace, practical skills-based assessment.

**Supplementary Information:**

The online version contains supplementary material available at https://doi.org/10.1007/s00405-026-10502-7.

## Introduction

 Ultrasound-guided fine-needle aspiration biopsy (FNAB) and core-needle biopsy (CNB) are cornerstone procedures in the diagnostic workup of head and neck pathology [1, 2]. Adequate operator competence is crucial for obtaining high-quality biopsy specimens and reliable diagnostic yield [3], and the growing number of otorhinolaryngologists performing their own biopsies underscores the need for robust and safe training programs.

Traditionally, training requirements have been defined by a minimum number of procedures or time spent in training. However, such volume-based standards do not account for individual learning curves and do not reliably ensure procedural competence [4, 5].

Consequently, competency-based assessment is increasingly advocated for training and certification in ultrasound-guided procedures [3, 4, 6, 7].

Competence assessment should encompass both theoretical knowledge and procedural performance [8]. Valid assessment tools exist for evaluating diagnostic head and neck ultrasound performance [6, 9, 10] and for testing the theoretical knowledge of ultrasound [11]. In contrast, no validated instrument specific to ultrasound-guided interventional procedures in the head and neck currently exists [4]. To address this gap, this study aimed to develop a multiple-choice question (MCQ) test to assess the theoretical knowledge required for safe and effective ultrasound-guided head and neck biopsies and to gather validity evidence supporting its use as the knowledge component of competency-based assessment.

## Materials and methods

### Study design, setting and validity framework

An MCQ test was developed and validity evidence was gathered using a contemporary version of Messick’s framework, which organizes evidence into five sources: content, response process, internal structure, relations to other variables, and consequences (Table 1) [12]. This framework aligns with recommendations from the American Educational Research Association and provides a robust method for accumulating validity evidence for new educational assessments.

**Table 1:** Summary of validity evidence for the MCQ test, aligned with Messick’s framework. This table outlines how each source of validity was addressed and the key findings supporting the test’s use

Source of Validity Evidence	Definition (Messick’s criteria)	Evidence from This Study
Content	The content of the test should measure the intended construct. Here, theoretical competence in head & neck ultrasound-guided biopsy.	**Expert Delphi consensus**: Test blueprint and items were developed based on comprehensive literature review and refined by an international panel of 7 experts through two Delphi rounds. All final items were rated highly relevant (mean relevance≥4/5).
Response Process	The integrity of the test administration and item response process is preserved. Scores are not unduly influenced by extraneous factors.	**Standardized administration**: All participants took the test via an identical online platform. Clear instructions were provided, and time was sufficient for all (no timing issues observed). The test items followed best-practice guidelines (single-best-answer MCQs), reducing cognitive bias or misinterpretation. There were no technical errors or reports of confusion during test taking.
Internal Structure	The test items demonstrate internal consistency and measure a single construct: similar scores would be obtained on re-testing (reliability).	**High internal consistency**: Cronbach’s α = 0.82 for the final 21-item test, indicating good reliability. Item analysis showed most items with positive corrected item-total correlations (median *r*=0.40). Two items with negative discrimination were removed, which improved α from 0.79 to 0.82.
Relation to Other Variables	Test scores correlate with other measures or groups as expected. Here, scores should reflect level of experience.	**Group differences**: As hypothesized, experienced clinicians scored significantly higher (mean 20.0 ± 0.9) than course participants (17.3 ± 2.8) and novices (13.5 ± 3.6), *p* < 0.001. Novices, course participants, and experts had clearly separated score distributions (ANOVA, *p* < 0.001). These known-groups comparisons confirm the test is sensitive to experience level.
Consequences	The implications of test scores and the pass/fail decision are appropriate: standard setting supports valid interpretation.	**Contrasting-groups’ standard setting**: A pass/fail cut score of 18 (86%) was determined. At this threshold, 100% of experts passed and 87% of novices failed, with only 8 novices false-positive. Course participant pass rate was 63%. These outcomes align with expectations, supporting the chosen cut score’s validity. These findings support interpretation of the test as a measure of biopsy-specific theoretical knowledge, but also indicate that the cut-score is sample-dependent and that additional validation against practical performance is needed before any high-stakes certification use.

The study was reviewed and deemed exempt from formal ethics committee approval due to its educational nature (F-25085416). It was conducted in accordance with the Declaration of Helsinki and local data protection regulations (Danish Data Protection Agency protocol (P-2024-16909).

This prospective validation study consisted of two main phases: (1) MCQ test development using a modified Delphi process, and (2) collection of additional validity evidence through cross-sectional test administration to groups with different experience levels.

The validation was conducted in Copenhagen, Denmark, in conjunction with the 5th Copenhagen International Head & Neck Ultrasound Course.

### Phase 1: test development via Delphi consensus

A modified Delphi method was employed to define essential content domains and items for the theoretical test. An international panel of seven experts in head and neck ultrasound and biopsy procedures was assembled, including five otolaryngology-head and neck surgeons and two radiologists representing United States (*n*=3), United Kingdom (*n*=2), Germany (*n*=1), and Taiwan (*n*=1). Experts were selected based on their leadership in the field, routine performance of ultrasound-guided head and neck biopsies, involvement in postgraduate ultrasound training, and a strong publication record.

The Delphi process was administered via REDCap online surveys, with anonymized responses to encourage candid feedback. This format granted all experts an equal opportunity to contribute independently and helped prevent individual dominance, thereby minimizing bias in the consensus process. Experts were informed that the test targeted knowledge domains specified in the EFSUMB Level 2 recommendations for head and neck ultrasound, which include ultrasound-guided interventions [4].

Before Delphi round one, the principal investigator (KAL) and steering committee (LK, JM, TKA, TT) conducted a targeted literature review and developed an initial item pool [1, 4, 13–16]. The steering committee comprised two otolaryngology-head and neck surgeons with expertise in head and neck ultrasound (HNUS), a senior consultant in head and neck pathology, and a professor in medical education.

In Delphi round 1, an invitation email was sent to the experts outlining the purpose and details of the study. This initial item pool of 26 questions adhered to established item-writing guidelines [17, 18]. Each item was single-best-answer format with three answer options (1 correct answer and 2 distractors) [19]. Three-option items were chosen because item-writing literature suggest that three-option MCQs often perform comparably to four-option items while reducing the number of non-functional distractors [19]. We knowledge, however that his format increases the probability of guessing the correct answer from 25% to 33%.

Experts rated each question’s relevance on a 5-point scale (1=not relevant, 3=relevant but not essential, 5=essential) and provided comments on wording clarity or content accuracy. They were also encouraged to suggest new questions or modifications and to recommend images/videos for inclusion if pertinent.

The steering committee predefined the inclusion criteria for items as a mean relevance rating of four or higher. If an item received a lower mean rating and revisions were suggested by the expert panel, the steering committee incorporated the proposed changes and resubmitted the revised item for the reevaluation in the subsequent Delphi round.

After round 1, the steering committee reviewed ratings and feedback. No items were excluded between the first and the second round. This was done to allow the experts to revise their opinion on questions from round 1 to round 2. Items were revised for clarity where needed. If more than 20% of experts proposed new items on overlapping topics, the steering committee categorized these suggestions and included them in the next round.

In Delphi round 2, experts re-rated the revised item set and new items. They were shown summary statistics from round 1 (distribution of ratings and anonymized comments) and asked to re-rate each item’s relevance and provide any further feedback. Consensus inclusion criteria were defined a priori to include items that had mean scores of ≥4.0 and ≥70% of experts rated 4 or 5 [20]. Consensus was reached after two rounds; therefore, a third round was not required. Examples of items are provided in **Appendix 1**.

### Phase 2: validity evidence and test administration

Phase 2 focused on collecting validity evidence from the remaining four sources by administering the MCQ to novices, course participants, and experienced clinicians. Novices (medical students at the University of Copenhagen) were recruited via a local social media group. Experienced clinicians were recruited from professional networks (senior consultants in Head and Neck surgery, Endocrinology and Radiology); this group was expected a priori to pass the test. Course participants completed the test at the end of day one of an international head and neck ultrasound (HNUS) course in Copenhagen.

All groups completed the MCQ online via a uniform interface accessed by survey link or QR code, with forward-only navigation to prevent skipping or revisiting items. No time limit was imposed. Each correct response earned 1 point, incorrect responses earned 0. Responses were anonymized.

## Participants

Three groups were recruited representing increasing expertise in ultrasound-guided head and neck biopsy:

- Novices: medical students with no prior head and neck ultrasound-guided biopsy experience.
- Course participants: residents and junior specialists in otolaryngology, endocrinology or radiology attending an international head and neck ultrasound course.
- Experienced clinicians: senior consultants in otolaryngology-head and neck surgery or endocrinology and radiology with extensive experience in diagnostic ultrasound and routine performance of FNAB/CNB.

## Statistics

Data were captured in REDCap (v13.7.14) and analyzed in IBM SPSS Statistics (v28). Significance level was set at *p*<0.05.

A Wilcoxon rank sum test was performed based on the specialty of the expert panel (otolaryngology–head and neck surgery vs. radiology/endocrinology).

To assess internal structure, we performed classical item analysis, item difficulty (proportion correct) and item discrimination (point–biserial correlation with total score) [21]. Items were classified a priori by difficulty/discrimination, poorly performing items were removed (see Table 2). Internal consistency reliability was calculated with Cronbach’s α: α≥0.80 was prespecified as acceptable for this knowledge test [22].

**Table 2:** Classification of MCQ items by difficulty and discrimination (initial 23-item test**).** Difficulty is the proportion of participants answering the item correctly. Discrimination is quantified by the point-biserial correlation between item score and total score

Classification	Item Difficulty	Item Discrimination (Point-Biserial)	Number of Items (*n*)
Level I (Optimal items)	0.45–0.75	> 0.20	*n*=8 (35%)
Level II (Acceptable items)	0.76–0.91	> 0.15	*n*=10 (43%)
Level III (Difficult items)	0.25–0.44	> 0.10	*n*=1 (4%)
Level IV (Extremely difficult or easy)	< 0.24 or > 0.91	*Any* (or poor discrimination)	*n*=4 (18%)

For validity evidence based on relations to other variables, total scores were compared across novices, course participants, and experienced clinicians using one-way ANOVA. Pairwise differences were examined with independent-samples t tests (Welch’s correction applied when variances were unequal, homogeneity checked by Levene’s test). Corresponding effect sizes (Cohen’s *d*) are reported.

To establish defensible consequences of the test, the contrasting groups’ standard setting method was used: novice and experienced subjects’ score distributions were plotted, and the intersection defined the pass/fail cut score [23]. Pass rates were then calculated by group, false negatives (experienced below the pass rate), and false positives (novices above the pass rate). The association between experience level and pass/fail outcome was tested with Fisher’s exact test. To quantify uncertainty around the contrasting groups’ standard setting method, we estimated a 95% confidence interval using nonparametric bootstrap resampling of the novice and experienced clinician score distributions (10.000 resamples).

An a priori power calculation for the MCQ validation was based on the novice-versus-expert comparison and anticipated an 8-point mean difference between novices and experts; with two-sided α=0.05 and 80% power, ≥16 participants per group were required [11, 24].

## Results

### Delphi process and test content

**Fig. 1:**
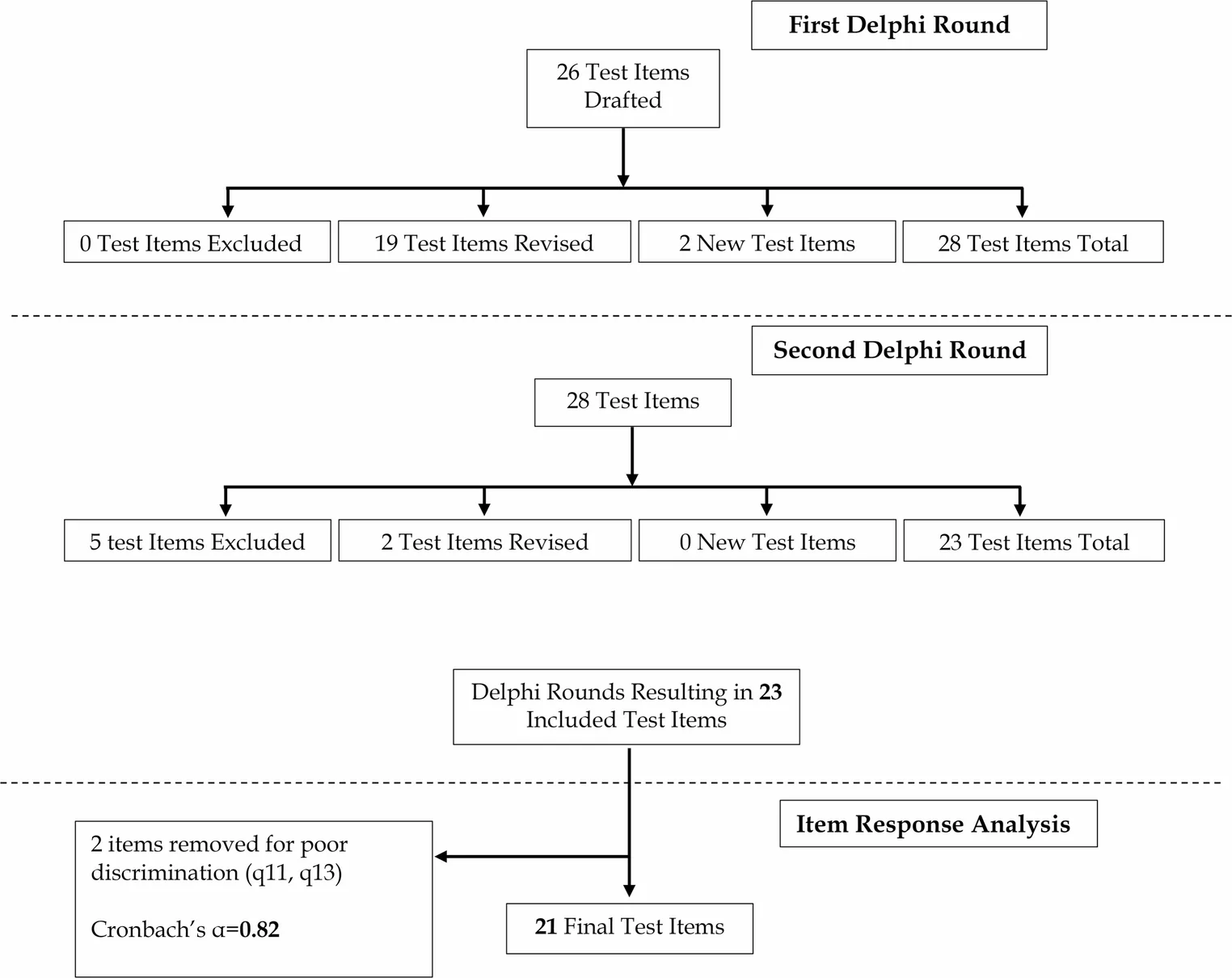
Flowchart of the Delphi process with excluded, revised, new, and included test items. 21 items were included after item response analysis based on data from the study with novices and experienced participants

Seven experts completed the two Delphi rounds and reached consensus on the MCQ content (Fig. 1). Round 1 confirmed the relevance of the drafted items, and experts’ suggestions added two new items. In round 2, the panel rated a total of 28 items; 23 met the a priori inclusion criteria and formed the initial test bank. Item-relevance rating did not differ by specialty (*p*=0.64).

### Item performance and internal consistency

In total, 103 participants completed the test (59 novices, 27 course participants, 17 experienced). Course participants had a mean of 1.7 years of prior head and neck biopsy experience (SD, 1.4; range, 0–5 years). Total scores on the 23-point scale ranged from 5 to 21. Eighteen of the initial 23 items (78%) were classified as Level I or II, indicating acceptable difficulty and good discrimination. One item was Level III, and four were level IV. Two of the level IV questions were easy knowledge questions that virtually all participants answered correctly (difficulty index>0.93 for items 4 and 8), and two were problematic items with poor discrimination (items 11 and 13). Following the item analysis, items 11 and 13 were removed, improving internal consistency from Cronbach’s α=0.79 to α=0.82. Corrected item-total correlations for the final 21 items ranged from 0.25 to 0.56, with a median of 0.40. Item classifications are summarized in Table 2.

### Relations to other variables

**Fig. 2:**
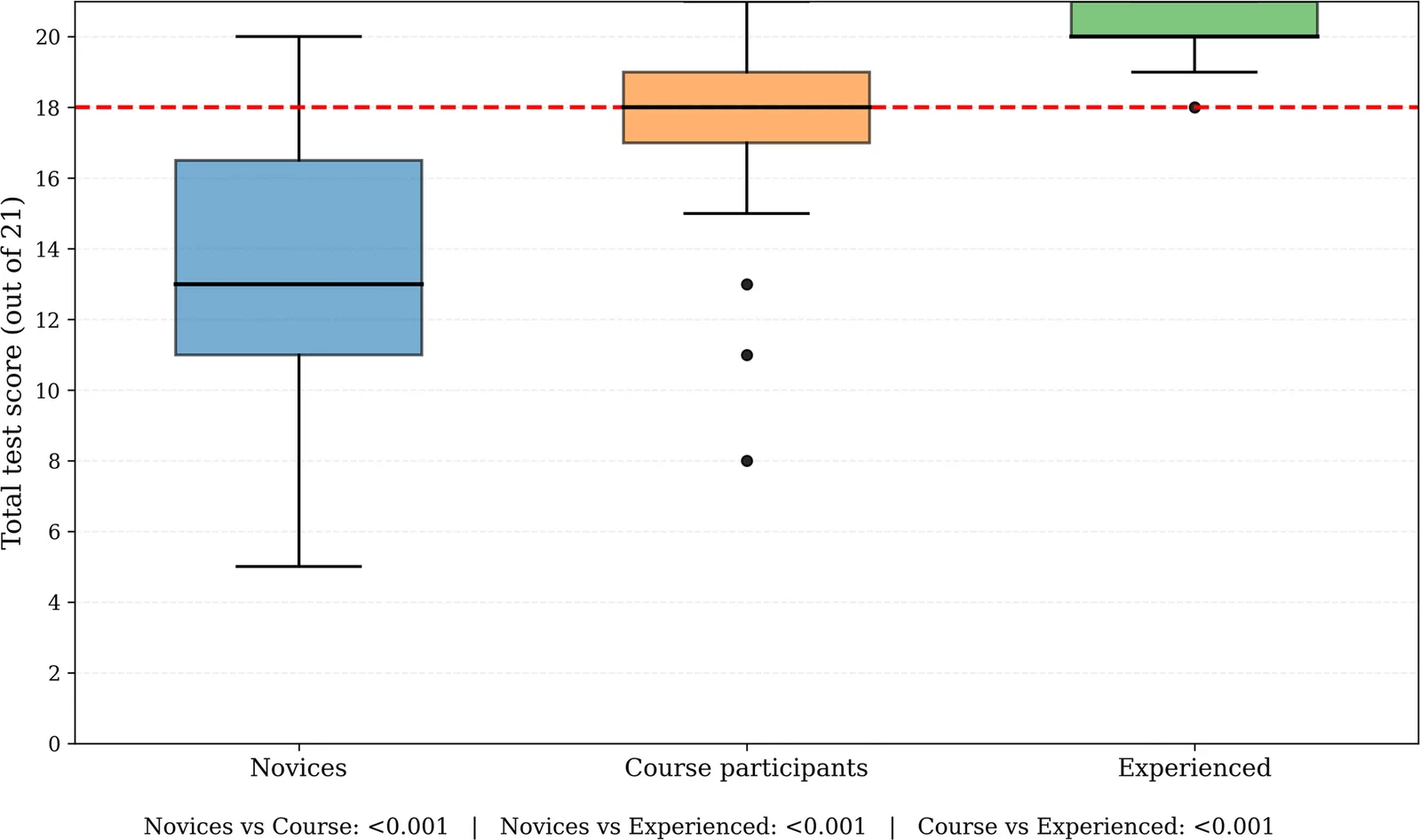
Multiple-choice test total scores by group, novices (*n* = 59), course participants (*n*=27), and experienced clinicians (n=17). Boxes show median and interquartile range, whiskers indicate range, points indicate outliers. The dashed red line marks the pass–fail cut-score. All pairwise comparisons were significant, p<0.001

MCQ scores increased with experience level (Fig. 2; Table 3). Mean (±SD) scores were 13.5±3.6 for novices, 17.3 ± 2.8 for course participants, and 20.0 ± 0.87 for experienced clinicians. One-way ANOVA showed a significant overall difference in scores among the three groups (*p*<0.001). Pairwise comparisons were all significant (*p*<0.001): experienced clinicians scored on average 6.5 points higher than novices (95% CI 4.7–8.2 points), and 2.7 points higher than the course participants (95% CI 1.3–4.2 *points*). The course participants outscored the novices by 3.8 points on average (95% CI 2.2–5.3). These large score gaps (all Cohen’s *d*>1.0) indicate the test has excellent discriminatory ability.

**Table 3:** Comparison of MCQ scores between experience levels. Mean scores (± SD) for each group are shown, along with absolute mean differences between groups. All pairwise differences were statistically significant (*p*<0.001)

Group Comparison	Mean Score (±SD)	Absolute Difference in Mean Score
Experienced (*n*=17) vs. Novices (*n*=59)	20.0 ± 0.9 vs. 13.5 ± 3.6	6.5 points
Experienced (*n*=17) vs. Course Participants (*n*=27)	20.0 ± 0.9 vs. 17.3 ± 2.8	2.7 points
Course Participants (*n*=27) vs. Novices (*n*=59)	17.3 ± 2.8 vs. 13.5 ± 3.6	3.8 points

### Consequences

**Fig. 3:**
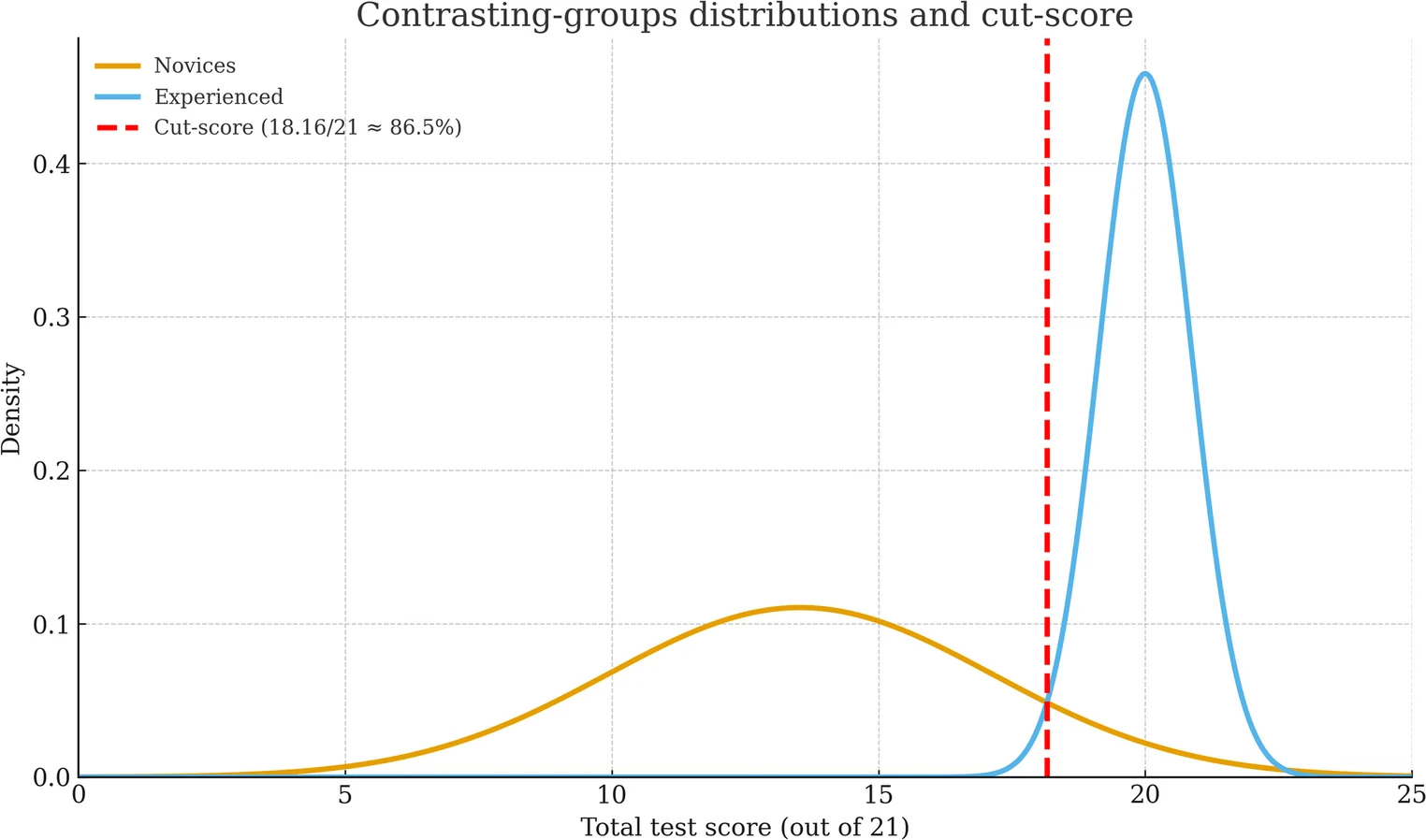
Multiple-choice test score distributions for novices (*n* = 59; yellow) and experienced clinicians (*n* = 17; blue). The pass–fail cut-score (18/21) was determined by the contrasting-groups’ standard setting method at the intersection of the two distributions (red)

The contrasting groups’ standard-setting method was used to establish a pass/fail cut-off score, at 18 points (out of 21). The observed continuous cut-score was 18.16, corresponding to an operational cut-score of 18/21, and the 95% confidence interval around the cut-score was 17.60 to 18.91. At this standard, 8/59 (13.6%) novices, 17/27 (63%) course participants, and 17/17 (100%) experienced clinicians passed (see Figs. 2 and 3). No experienced participant fell below the cut (false negative), and eight novices scored above the cut (false positives). Experience level was strongly associated with pass/fail outcome (*p*<0.001).

## Discussion

We developed an MCQ test based on expert consensus to assess theoretical knowledge relevant to ultrasound-guided head and neck biopsies. The final test showed high internal consistency, differentiated between levels of expertise, and yielded a defensible pass/fail standard that can be incorporated into competency-based assessment.

A key strength of the study is the collection of validity evidence across all five sources in Messick’s framework. Content evidence was supported by international expert consensus, response-process evidence by standardized online administration with uniform instructions and forward-only navigation, internal-structure evidence by item statistics and reliability calculations, and relations-to-other-variables evidence by stepwise score increases with experience and large effect sizes. Consequence evidence was provided by the standard setting process and observed classification patterns; importantly, the chosen cut score produced no false negatives among experienced clinicians in our cohort.

Our findings align closely with established approaches to ultrasound knowledge-test development that combine expert consensus, psychometric analysis, and known-groups comparisons [11, 25–27]. Compared with other MCQs [11, 25], our test is intentionally brief to facilitate use in educational setting and competency-based assessment workflow. Although shorter tests can reduce reliability, our internal consistency remained above commonly used thresholds for knowledge tests [18], and the score differences between groups were large enough to support meaningful decisions. An additional distinguishing feature is the domain specificity of our test. Whereas most ultrasound knowledge tests focus on general diagnostic skills, physics, or image interpretation, our MCQ test targets interventional biopsy-specific knowledge. This emphasis addresses a practical gap, given the growing use of ultrasound-guided interventions and their inclusion in advanced training recommendations [4].

The course participants represented an intermediate and heterogeneous learner group and completed the MCQ during, rather than after, their ultrasound training. Full competence was therefore not expected in all participants. The finding that 17/27 (63%) passed the test is thus consistent with the expected variation in prior experience and remaining knowledge gaps within this group. In routine educational practice, this supports the use of the test as a formative or complementary assessment to identify learners who may benefit from further theoretical preparation before progression to independent biopsy practice.

Several limitations warrant attention. First, the Delphi panel comprised seven experts. A larger panel might have yielded different item selection and weighting, and prior Delphi-based ultrasound studies have often included more experts [6]. Small panels can narrow perspectives and increase the influence of individual priorities. We mitigated this risk through international representation across multiple countries and specialties.

Second, the experienced group in phase 2 was modest in size and drawn from a limited geographical and training context, which may limit generalizability across health systems, languages, and curricula. Although the a priori sample size target for the novice-versus-expert comparison was met, the unequal group sizes and the relatively small experienced clinician group may still reduce the precision and stability of variance estimates and may limit generalizability. Future studies should examine the test’s performance in international cohorts across different specialties, healthcare systems, and training programs.

The cut-score should be interpreted with caution. Although the contrasting-groups method supported an pass/fail threshold of 18/21, the confidence interval around the cut-score suggested some sample sensitivity, particularly given the small experienced group and the unequal variances across groups. Furthermore, 8 of 59 novices (13.6%) scored above the cut-score, indicating that the MCQ should not be used as a stand-alone high-stakes certification instrument at this stage. Instead, it should be used as part of a broader programme of assessment and combined with an objective assessment of procedural performance to reduce the risk of passing candidates with sufficient theoretical knowledge but insufficient practical competence.

Third, we did not link MCQ performance to observed biopsy performance or clinical outcomes. Without correlation to a practical skills assessment or patient-level endpoints, we cannot yet claim criterion-related evidence connecting knowledge scores to procedural competence.

Fourth, the very high scores and narrow variance among experienced clinicians indicates a ceiling effect, suggesting that the current version of the test may be less sensitive for differentiating among highly experienced clinicians. However, as the aim of the test is to provide feedback to trainees and aid in their certification, we can accept that it is not suitable to differentiate levels of very experienced clinicians.

Beyond the scope of our own data, existing literature underscores the synergy between theoretical knowledge and procedural skill. For example, Kohls-Gatzoulis et al. demonstrated that teaching cognitive skills in a surgical course significantly improved subsequent technical performance [28], consistent with Miller’s framework positing knowledge as the foundation of clinical competence [29]. This suggests that a robust theoretical understanding can enhance and complement practical skill execution. This interplay is further supported by emerging evidence in ultrasound education: a recent systematic review identified domain knowledge as a significant determinant of point-of-care ultrasound proficiency [30], and a meta-analysis found concurrent improvements in both ultrasound knowledge and hands-on procedural skills [31].

Importantly, the present instrument assesses theoretical knowledge only and should not be interpreted as a comprehensive measure of biopsy competence. Competence in ultrasound-guided biopsy includes both cognitive and procedural components [8, 29]. The MCQ therefore addresses the cognitive foundation for safe performance but cannot substitute for direct assessment of technical skill, decision-making in practice, or patient-level outcomes.

In conclusion, this study provides validity evidence for a concise MCQ test assessing biopsy-specific theoretical knowledge in ultrasound-guided head and neck FNAB and CNB. The instrument supports the knowledge component of competency-based assessment and could be used together with validated practical skills assessments to provide structured feedback during training and aid certification decisions.

## Supplementary Information

Below is the link to the electronic supplementary material.

Supplementary Material 1
