# Supplementary material for: Development and validation of a theoretical test for ultrasound-guided biopsy competence in the head and neck
Source: Eur Arch Otorhinolaryngol. 2026 Aug 3;283(11):7387–95. doi: 10.1007/s00405-026-10502-7 (PMC13748091; doi:10.1007/s00405-026-10502-7)
Supplement: Supplementary file 1 [file 405_2026_10502_MOESM1_ESM.docx]

## **Appendix 1.** Example MCQ items

**Item X**
Which needle gauges are most commonly used during an ultrasound-guided fine-needle aspiration biopsy of a thyroid nodule?

A. 18–22 G (1.9–1.2 mm)
B. 23–27 G (0.8–0.5 mm)
C. 28–32 G (0.45–0.31 mm)

**Item Y**
What is the first thing to do if visualization of the biopsy needle is lost during an ultrasound-guided procedure?

A. Keep the needle still and increase the frequency
B. Keep the needle still and decrease the gain
C. Keep the needle still and tilt the transducer

**Item Z**
What is a possible advantage of using a non-aspiration (capillary) technique compared with the aspiration technique in fine-needle aspiration cytology (FNAC)?

A. It provides a better preservation of the cellular morphology
B. It decreases the likelihood of obtaining a bloody sample
C. It is more comfortable for the patient

**Item A**
How should the bevel of the needle be oriented to improve visualization of the needle tip on ultrasound?

A. It does not matter
B. Towards the transducer
C. Away from the transducer

**Item B**
Core-needle biopsy has emerged as a complementary method to FNAB and an alternative to open surgical biopsy. When firing the stylet to reach the target lesion with a semi-automated advancing core-needle device, there is a risk of inadvertent injury to structures located ___ to the target lesion:

A. Distal/deep
B. Proximal/superficial
C. Both
